# A Case of Necrotizing Pneumonia Complicated by Hydropneumothorax

**DOI:** 10.7759/cureus.57775

**Published:** 2024-04-07

**Authors:** Asim Alawad, Muhnad Mohamed Abdeen, Khalid Y Fadul, Moayad A Elgassim, Shahda Ahmed, Mohamed Elgassim

**Affiliations:** 1 Emergency Medicine, Hamad Medical Corporation, Doha, QAT; 2 Medical Education, Hamad General Hospital, Doha, QAT; 3 Emergency Medicine, Hamad Medical Corporation, Doha, Qatar, QAT; 4 Emergency Medicine, Hamad General Hospital, Doha, QAT

**Keywords:** cavitation pneumonia, emergency medicine physician, complication, hydropneumothorax, nercrotizing pneumonia

## Abstract

We present a case of a 58-year-old man who had asthma and developed necrotizing pneumonia (NP). The Computed Tomography (CT) scan of his chest showed cavitating consolidation with hydropneumothorax. Despite chest tube insertion and antibiotics, the patient did not improve. Therefore, surgical intervention was considered, and he underwent a right posterior-lateral thoracotomy, with middle lobe lobectomy, and decortication. As a result, the patient's condition started to improve, and he was discharged in good health.

## Introduction

Necrotizing pneumonia (NP) is a severe and uncommon type of pneumonia that causes abscesses and cavitation to form within the lung tissue [1]. The most common etiology of NP includes *Staphylococcus aureus*, *Streptococcus pneumoniae*, and *Klebsiella pneumoniae* [1]. Community-acquired pneumonia presenting as necrotizing pneumonia is rare and life-threatening [2]. We want to present a rare case of NP, which was further complicated by hydropneumothorax.

## Case presentation

**Figure 1:**
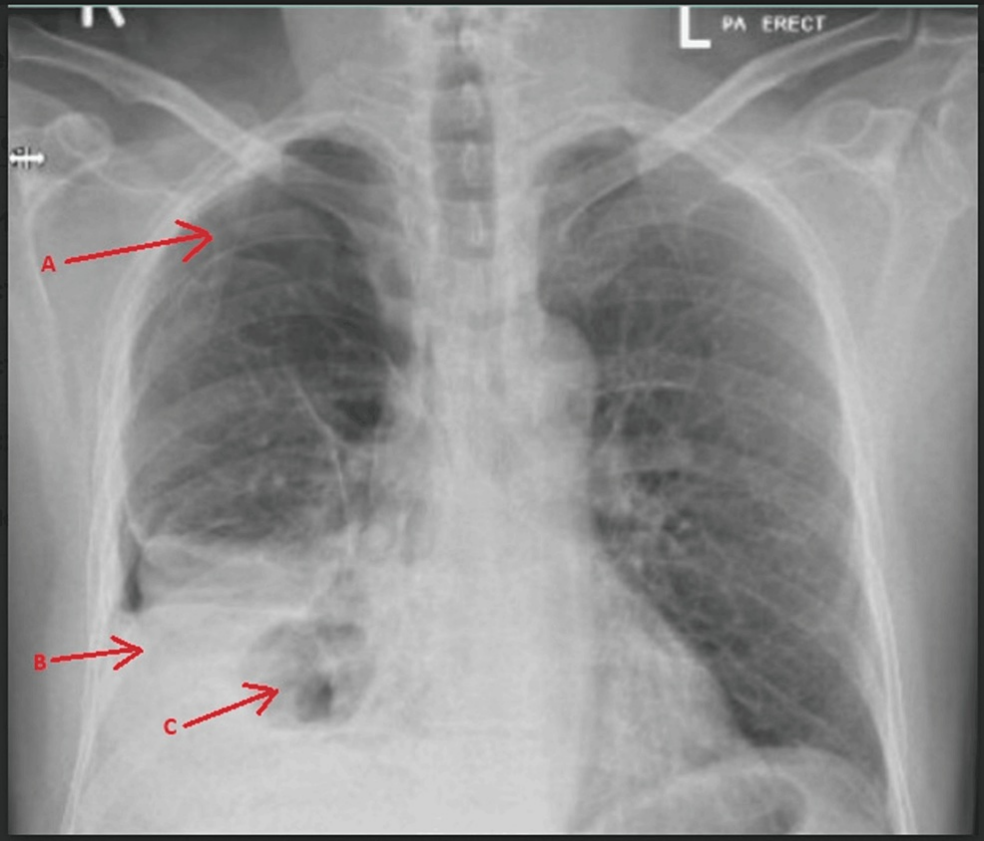
Chest X-ray Right hydropneumothorax with collapse of the right lung (arrow: A). Consolidation is noted in the mid and lower zones of the right lung (arrow: B). A cavitating lesion was noted in the lower zone of the right lung (arrow: C).

A 58-year-old male, with bronchial asthma presented to the Emergency Department with shortness of breath and right lower chest pain, associated with a dry cough for 6 days. Initially, his vitals were stable, a white blood cells of 8.6 (Reference range: 4-10 ×10^9^/L) and a C-reactive protein of 250 (Reference range: 0-5 mg/L). Chest X-ray (Figure 1) showed right hydropneumothorax with collapse of the right lung, along with consolidation and a suspected cavitating lesion. 

**Figure 2:**
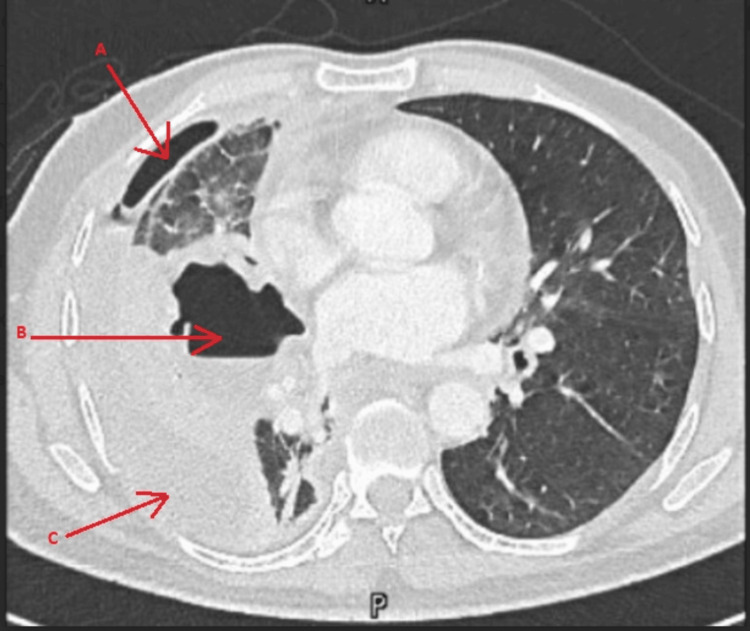
Axial CT scan Multiloculated pleural collection/pneumothorax (arrows: A & C), and cavitating consolidation in the right middle lobe (arrow: B)

**Figure 3:**
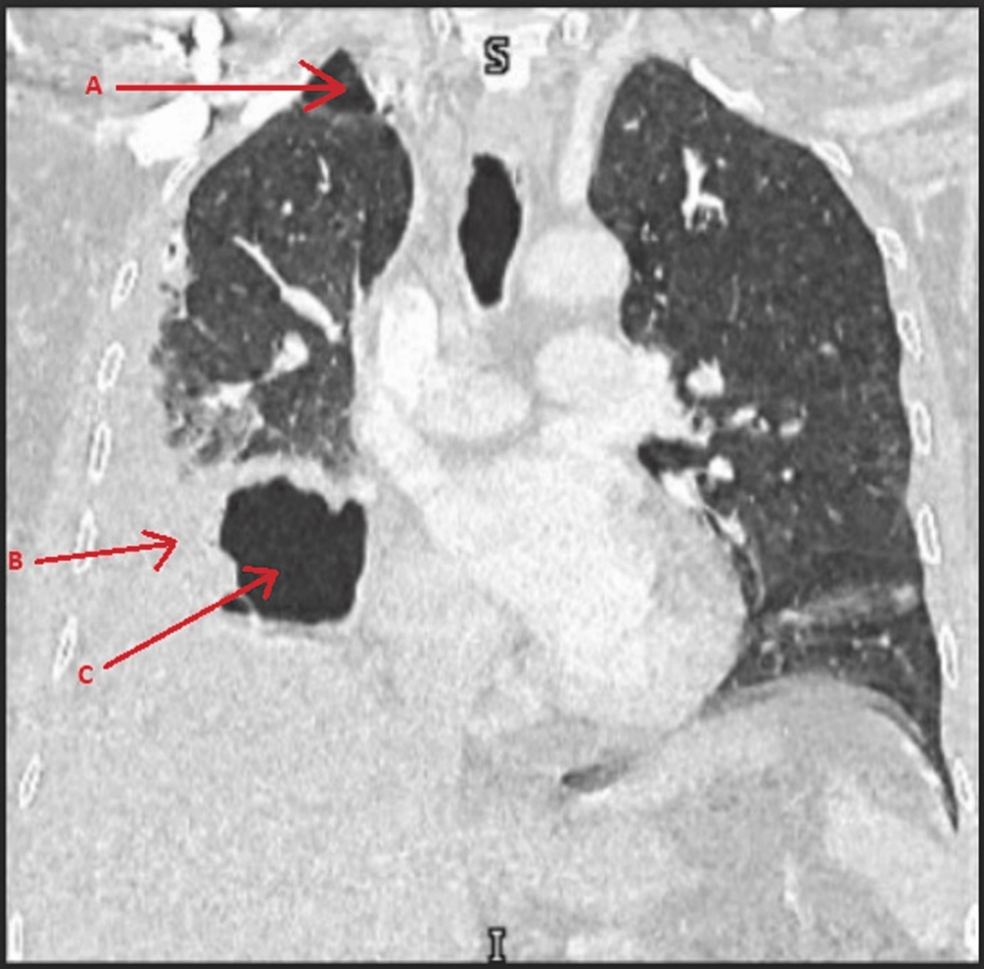
Coronal CT scan Multiloculated pleural collection/pneumothorax (arrows: A & B), and cavitating consolidation in the right middle lobe /collapse of the right lower lobe (arrow: C).

The patient became tachycardic and tachypneic, so a decision was made to insert a chest tube to drain the hydropneumothorax for a concern of sepsis. The fluid was brownish and turbid. The fluid was sent for culture, a Gram stain was done, and broad-spectrum antibiotics started. The patient did not improve clinically and on day 4, a CT scan of the chest was done (Figures 2, 3), which showed a cavitating consolidation in the right middle lobe, consolidation /collapse of the right lower lobe, and multiloculated pleural collection/pneumothorax as described.

On day 5, thoracic surgery was consulted and they took the patient for surgery on day 7. A right posterio-lateral thoracotomy, middle lobe lobectomy, and decortication were performed. The patient improved after the surgery and was discharged on day 15 with a plan of 2 weeks of ceftriaxone followed by oral amoxicillin-clavulanic acid. He continued to show improvement on outpatient follow-up.

## Discussion

NP, also known as cavitating pneumonia [2], is a rare but life-threatening complication of community-acquired pneumonia [2, 3]. It is a severe form of lung disease that can range from lung abscess to pulmonary gangrene [3, 4]. Common complications associated with it include bronchopulmonary fistula, pyopneumothorax, or parapneumonic effusion [1, 2, 4, 5]. In rare cases, it can also lead to extrapulmonary complications such as septic arthritis [5]. *Staphylococcus aureus*, *Streptococcus pneumoniae*, and *Klebsiella pneumoniae* are the most common causes of NP. The pathophysiology of NP is not well defined, but it is believed to be related to the inflammation process caused by toxins produced by the pathogen or due to associated thrombosis or vasculitis [1]. In our case, culture samples from the chest drain showed a polymicrobial growth, including *Streptococcus constellatus*, *Actinomyces turicensis*, and *Bulleidia extructa*.

A contrast-enhanced computed tomography of the thorax is considered the most reliable method for diagnosing NP. This is because it can help characterize NP, where the normal lung tissue is replaced by multiple cavities filled with either air or fluid. Chest radiographs may also show protrusion of the fissures due to the high intensity of the present inflammatory exudation [3]. However, it is difficult to diagnose NP using only clinical examination and serial chest radiographs as it cannot be differentiated from simple lobar pneumonia [2]. The value of CT thorax extends to detecting complications associated with NP as well [1, 2]. Bronchial and alveolar necrosis, along with air-filled cavities, are common features of NP. Pneumothorax or hydropneumothorax can occur as a complication of an existing bronchopulmonary fistula if the necrosis is near the pleura [4]. In our patient, the CT scan was preceded by a chest X-ray which showed hydropneumothorax with collapse, consolidation, and cavitary lesions. A definitive diagnosis was made through the CT scan.

The primary treatment for NP is supportive care and appropriate antibiotic therapy. In case the patient's condition does not improve, surgical intervention may be necessary to save their life [3]. Unfortunately, the patient's condition did not improve, and surgical intervention was required. There are two main types of surgical intervention: management of pleural disease and management of progressive parenchymal necrotizing infection. In cases of acute necrotizing lung infection with massive hemoptysis and pulmonary gangrene, surgical resection of the lung parenchyma may be necessary. This is particularly relevant in the context of acute necrotizing lung infection.

Patients with septic pulmonary embolism often have poor health and may be at high risk for surgery due to inadequate blood flow and low oxygen levels. Additionally, these patients often have acute lung injury and systemic inflammation, which can worsen during surgery [3]. The primary objective of the surgery is to manage the sepsis, drain the empyema, clear any lung abscesses, remove necrotic debris, and safeguard the unaffected lung from any potential complications [3]. Our patient improved after the surgical intervention and was discharged on antibiotic therapy. Subsequent follow-ups revealed significant improvement. 

## Conclusions

NP is an acute and rare type of pneumonia that leads to abscesses and cavitation to form within the lung tissue. It is important to consider NP when there is hydropneumothorax on the initial chest X-ray or a lack of improvement with chest drainage or antibiotics. Early CT scans and involving the thoracic surgery team are also recommended.
